# A deep learning framework for predicting disease-gene associations with functional modules and graph augmentation

**DOI:** 10.1186/s12859-024-05841-3

**Published:** 2024-06-14

**Authors:** Xianghu Jia, Weiwen Luo, Jiaqi Li, Jieqi Xing, Hongjie Sun, Shunyao Wu, Xiaoquan Su

**Affiliations:** College of Computer Science and Technology, Qingdao University, Qingdao, 266071 Shandong China

**Keywords:** Gene-disease associations, Deep learning, Graph augmentation, Protein complexes, Graph neural networks

## Abstract

**Background:**

The exploration of gene-disease associations is crucial for understanding the mechanisms underlying disease onset and progression, with significant implications for prevention and treatment strategies. Advances in high-throughput biotechnology have generated a wealth of data linking diseases to specific genes. While graph representation learning has recently introduced groundbreaking approaches for predicting novel associations, existing studies always overlooked the cumulative impact of functional modules such as protein complexes and the incompletion of some important data such as protein interactions, which limits the detection performance.

**Results:**

Addressing these limitations, here we introduce a deep learning framework called ModulePred for predicting disease-gene associations. ModulePred performs graph augmentation on the protein interaction network using L3 link prediction algorithms. It builds a heterogeneous module network by integrating disease-gene associations, protein complexes and augmented protein interactions, and develops a novel graph embedding for the heterogeneous module network. Subsequently, a graph neural network is constructed to learn node representations by collectively aggregating information from topological structure, and gene prioritization is carried out by the disease and gene embeddings obtained from the graph neural network. Experimental results underscore the superiority of ModulePred, showcasing the effectiveness of incorporating functional modules and graph augmentation in predicting disease-gene associations. This research introduces innovative ideas and directions, enhancing the understanding and prediction of gene-disease relationships.

**Supplementary Information:**

The online version contains supplementary material available at 10.1186/s12859-024-05841-3.

## Introduction

Gene mutations or genetic abnormalities play a pivotal role in the pathogenesis of various diseases. Consequently, uncovering the associations between genes and diseases is imperative to elucidate the underlying molecular mechanisms and enhance healthcare. While linkage analysis and genome-wide association studies are capable of detecting biomarkers, such as single nucleotide polymorphisms (SNPs), by examining genetic variations within human populations, these approaches are time and resource-intensive due to the necessity of analyzing numerous false positives [1]. Moreover, these methods primarily focus on direct connections between genotypes and phenotypes, thereby overlooking the complex interactions between molecules [2].

Recent years, computational methods rooted in molecular networks have emerged as a prominent approach to complement and enhance linkage analysis and genome-wide association studies, providing valuable insights into disease gene prediction [3–5]. The primary objective is to extract topological features that precisely capture the intricate connections between genes and diseases, including measures of topological similarity between genes and diseases [6–8], as well as other artificially extracted features [9–11]. Notably, graph embedding methods such as node2vec and graph neural networks like graph convolutional network (GCN) have witnessed extensive application in gene-disease association mining, showcasing commendable performance by automatically discovering potent latent features [12, 13]. Despite significant strides in existing research, certain issues impede detection performance, including the oversight in investigating cooperative relationships among molecules. For instance, in cellular activities, proteins often depend on collaborative interactions within protein complexes to execute specific functions [14]. Additionally, the effectiveness of disease gene prediction faces substantial hindrance due to the incompleteness of existing molecular networks, notably the protein interaction network, which lacks experimental validation for numerous interactions.

**Fig. 1:**
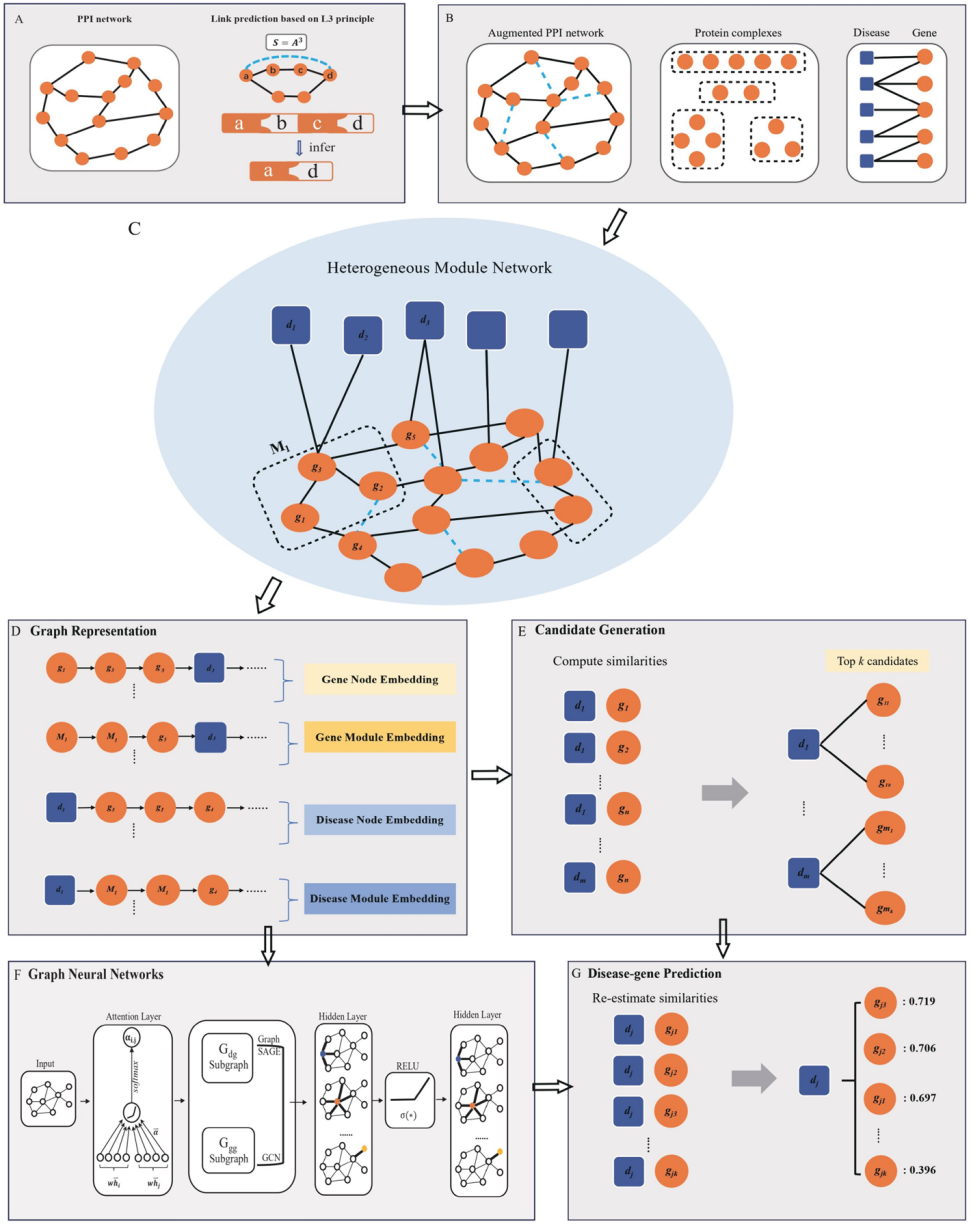
An overview of our proposed approach. Firstly, Data augmentation was performed on the protein–protein interaction (PPI) network with L3 principle (**A**). Then, by integrating augmented PPI network, protein complexes and disease-gene associations (**B**), a heterogeneous module network was built (**C**). Subsequently, initial low-dimensional embeddings were obtained by graph representation (**D**) for the heterogeneous module network and candidate genes were generated for each disease (**E**). Furthermore, a graph neural network was constructed to learn better representations by collectively aggregating information from topological structure (**F**). Finally, for each disease, the candidate genes were scored and re-ranked based on the embeddings generated by the graph neural network (**G**)

This paper introduces a novel paradigm centered on modules to encapsulate cooperative relationships among molecules, particularly focusing on protein complexes. We present ModulePred, an advanced deep learning framework designed for the purpose of mining gene-disease associations. To tackle the issue of data incompleteness, we initiate the process by conducting data augmentation on the protein interaction network through L3-based link prediction algorithms (Fig. 1A). L3-based link prediction algorithms integrate biological motivations into the prediction of protein–protein interactions, surpassing the performance of general-purpose algorithms [15]. Subsequently, the establishment of a heterogeneous module network (Fig. 1C) unfolds, seamlessly integrating disease-gene associations, augmented protein interactions, and protein complexes (Fig. 1B). Within this framework, a sophisticated graph embedding method is devised to harness the cooperative relationships intrinsic to the heterogeneous module network (Fig. 1D), subsequently deploying this method to generate candidate genes for each disease (Fig. 1E). Furthermore, a graph neural network is engineered to glean enhanced representations by collectively aggregating information from the topological structure (Fig. 1F). Ultimately, low-dimensional disease and gene embeddings are harnessed for gene prioritization (Fig. 1G).

## Materials and methods

### Graph data augmentation based on L3 principle

Even with significant advancements in high-throughput mapping techniques, a considerable number of human protein–protein interactions (PPIs) remain unknown compared to those that have been experimentally documented [16]. Network-based link prediction algorithms are gaining momentum as valuable computational tools for predicting undetected interactions. Such state-of-the-art algorithms rely on the triadic closure principle, which assumes that the number of paths of length two between two nodes is correlated with the likelihood of them also being directly connected. However, the triadic closure principle inadequately characterize PPIs, thereby failing to guarantee the correctness and reliability of predictions. Figure 1A illustrates that protein a and protein c share a path of length 2, indicating a potential interaction based on the triadic closure principle. PPIs often require complementary interfaces [17, 18]. As a result, protein a and protein c exhibit similar interfaces, as illustrated by their identical shapes in Fig. 1A. It is notable that such an interface does not typically guarantee that protein a and protein c interact with each other [15].

To address the aforementioned issue, Kovács et al.[15] proposed a novel link prediction predictor based on the L3 principle, positing that proteins linked by multiple paths of length three are more likely to have a direct link. As shown in Fig. 1A, an additional interaction partner of protein c (protein d) and protein a have a complementary interface, suggesting a possible direct interaction. Such an interaction can be predicted by using paths of length three (L3). In this paper, we adopted the L3 principle to perform data augmentation on the protein interaction network. Three L3 scores are assigned to each node pair, *x* and *y* (Eqs. 1–3)

1 $$\begin{array}{*{20}l} {L_{3}^{{CN}} \left( {x,y} \right) = \sum\limits_{{u,v}}^{{}} {a_{{xu}} a_{{uv}} a_{{vy}} } } \\ \end{array}$$

2 $$\begin{array}{c}{L}_{3}^{RA}\left(x,y\right)=\sum\limits_{{u,v}}^{{}}{a}_{xu}{a}_{uv}{a}_{vy}\left(\frac{1}{{k}_{u}}+\frac{1}{{k}_{v}}\right)\end{array}$$

3 $$\begin{array}{c}{L}_{3}^{AA}\left(x,y\right)=\sum\limits_{{u,v}}^{{}}{a}_{xu}{a}_{uv}{a}_{vy}\left(\frac{1}{log{k}_{u}}+\frac{1}{log{k}_{v}}\right)\end{array}$$

where ${k}_{u}$ represents the degree of node *u* while ${a}_{xu}$ is a binary variable. ${a}_{xu}=1$ if node *x* interacts with node *u* interacts, otherwise ${a}_{xu}=0$. ${L}_{3}^{RA}$ And ${L}_{3}^{AA}$ are degree-normalized versions of ${L}_{3}^{CN}$, derived from the insights obtained from RA (Resource Allocation) and AA (Adamic-Adar) [19]. When performing data augmentation, taking protein *x* as an example, first calculate similarity scores with all remaining nodes (excluding those already connected to *x*). Then, select the top *l* nodes with the highest similarity to *x* for ${L}_{3}^{CN}$, ${L}_{3}^{RA}$, and ${L}_{3}^{AA}$ respectively. The selected node sets are denoted as ${S}_{CN}$, ${S}_{RA}$, and ${S}_{AA}$. Lastly, create edges between *x* and each node in the set ${S=S}_{CN}\cup {S}_{RA}\cup {S}_{AA}$.

### Graph representation for the heterogeneous module network and candidates generation

As illustrated in Fig. 1C, a heterogeneous module network, denoted as $G=(V,E)$, was constructed by integrating disease-gene associations, augmented protein–protein interactions, and protein complexes (Fig. 1B). In this network, the node set *V*, consists of disease and gene nodes, with $V={V}_{d}\cup {V}_{g}$. And the edge set* E*, includes disease-gene associations and protein–protein interactions, $E={E}_{dg}\cup {E}_{gg}$. For simplicity, protein nodes are referred to as gene nodes, and protein interactions are represented as gene interactions. Certain nodes, such as *x* and *y*, exhibit cooperative relationships and belong to a module, denoted as ${M}_{1}$. This can be expressed as $x\in {M}_{1}$, $y\in {M}_{1}$, or ${M}_{1}=\{x,y\}$. ${M}_{1}$ is a member of the module set *M* that comprises of protein complexes.

In this study, Node2vec [20], a prevalent network embedding algorithm, was introduced to extract low-dimensional node representations from the heterogeneous module network. Firstly, we utilized random walks to generate multiple neighbor sequences for each node. It should be noted that two types of sequences were generated for each node: the conventional node sequences ${Q}^{n}$ and enhanced sequences ${Q}^{m}$ that incorporate both nodes and modules. As depicted in Fig. 1D, the sequence ${q}_{1}^{n}={g}_{1}\to {g}_{3}\to {g}_{5}\to {d}_{3}\dots$ is a walk sequence starting from ${g}_{1}$ that only contains node. By replacing gene nodes with their corresponding module numbers (both ${g}_{1}$ and ${g}_{3}$ belong to${M}_{1}$, so they are both replaced with${M}_{1}$), the sequence ${q}_{1}^{n}$ can be transformed into ${{q}_{1}^{m}=M}_{1}\to {M}_{1}\to {g}_{5}\to {d}_{3}\dots$. Here, ${q}_{1}^{n}\in {Q}^{n}$ and${q}_{1}^{m}\in {Q}^{m}$. Then, all the sequences of ${Q}^{n}$ were treated as texts, where nodes were considered as words, and the skip-gram model, a typical natural language processing model, was applied to learn the node embeddings. Similarly, all the sequences of ${Q}^{m}$ were provided to the skip-gram model to learn the module embeddings. If a node does not belong to any module, its node embeddings were used as its module embeddings.

For each disease, we computed cosine similarities between its node embedding and the embeddings of all gene nodes. Then, we selected the top-*k* genes with the highest similarity as candidates for each disease (Fig. 1E). In the disease gene prediction stage, we focused only on calculating similarities between each disease and its candidate genes, significantly reducing the computational complexity.

### Graph neural networks for the heterogeneous module network

A graph neural network was constructed based on the graph representation, aimed at improving the learning of low-dimensional node representations by aggregating information from the topological structure. The embedding vectors obtained from the graph representation served as initial node features for the graph neural network. In the graph neural network architecture (Fig. 1F), a graph attention network was initially employed to assign different weights to neighbors for updating node information. Subsequently, two graph convolutional layers were applied to protein interactions, while two GraphSage layers were used for disease-gene associations.

The heterogeneous module network employed the Graph attention network (GAT) to compute the hidden states of each node through a self-attention strategy. This can be defined by Eqs. 4 and 5:

4 $$\begin{array}{*{20}l} {H_{i}^{1} = \sum\limits_{{j \in N_{i} }}^{{}} {\alpha _{{i,j}} W^{0} H_{j}^{0} } } \\ \end{array}$$

5 $$\begin{array}{c}{\alpha }_{i,j}={{softmax}_{j}({e}_{ij})=softmax}_{j}(LeakyReLU({\overrightarrow{a}}^{T}[{W}^{0}{H}_{i}^{0}||{W}^{0}{H}_{j}^{0}]))\end{array}$$

where *N*_*i*_ represents the neighborhood set of node *i*, ${W}^{0}$ is a trainable weight matrix, ${H}_{j}^{0}$ is the initial features of node *j* obtained from graph representation, and ${H}_{i}^{1}$ denotes the embedding vector of node *i* obtained by GAT. A shared attentional mechanism $a: {}^{ F}\times {}^{ F}\to$ (*F* represents the number of the node features output by the layer) is performed on the nodes to compute attention coefficients ${e}_{ij}=a({W}^{0}{H}_{i}^{0},{W}^{0}{H}_{j}^{0})$ that represent the importance of node *j* ‘s features to node *i*. ${\alpha }_{i,j}$ is calculated by normalizing ${e}_{ij}$ with the softmax function. $\overrightarrow{a}$ is a weight vector to parameterize the single-layer feedforward neural network that forms the attention mechanism a. $[{W}^{0}{H}_{i}^{0}||{W}^{0}{H}_{j}^{0}]$ signifies the concatenation of ${W}^{0}{H}_{i}^{0}$ and ${W}^{0}{H}_{j}^{0}$, and $LeakyReLU$ is the activation function. Specifically, ${H}_{j}^{0}=[{H}_{j}^{node}||{H}_{j}^{module}]$, where ${H}_{j}^{node}$ and ${H}_{j}^{module}$ represent the node embedding and module embedding of node *j* obtained from the graph representation, respectively.

For the subgraph ${G}_{gg}$, the convolution operation was conducted by the graph convolutional layer. Graph convolutional layer can be defined as *Eq. *6:

6 $$\begin{array}{*{20}l} {H_{i}^{{k + 1}} = \sigma \left( {\sum\limits_{{j \in N_{i} }}^{{}} {\frac{1}{{c_{{ji}} }}} H_{j}^{k} W^{k} + b^{k} } \right)} \\ \end{array}$$

where ${c}_{ji}=\sqrt{|{N}_{j}|}\times \sqrt{|{N}_{i}|}$, ${b}^{k}$ is a trainable bias matrix, and ${W}^{k}$ is a trainable weight matrix. The activation function $\sigma$, set as RELU in this paper, is applied to the layer. ${H}_{j}^{k+1}$ ($k\ge 1$) represents the embedding vector of node *j* in the *k* + *1*_*th*_ layer, and ${H}_{j}^{1}$ captures the information of node *j* obtained by GAT.

GraphSage layer was adopted for the subgraph ${G}_{dg}$. In contrast to the graph convolutional layer that utilizes the full neighborhood set, GraphSage layer samples a specific proportion of neighbors to aggregate information. The embedding process of GraphSAGE is defined by Eqs. 7 and 8:

7 $$\begin{array}{c}{H}_{{N}_{i}^{\prime}}^{k+1}={AGG}_{k+1}\left(\left\{{H}_{j}^{k},\forall j\in {N}_{i}^{\prime}\right\}\right)\end{array}$$

8 $$\begin{array}{c}{H}_{i}^{k+1}=\sigma ({W}^{k+1}\cdot [{H}_{i}^{k}||{H}_{{N}_{i}{\prime}}^{k+1}])\end{array}$$

where ${N}_{i}{\prime}$ represents a subset from the neighborhood set *N*_*i*_. The aggregation function, denoted as ${AGG}_{k+1}$, was chosen as the mean aggregator in this study, and hence GraphSage takes the mean over neighbors of node *i* according to Eq. 7. Different with the graph convolutional layer, GraphSAGE concatenates the node representation with the mean aggregation of neighbor nodes as shown in Eq. 8, which avoids node information loss.

The outputs of the various convolutional layers were aggregated to incorporate information from all types of edges for each node. In this study, two layers were constructed for GCN and Graphs sage, which has demonstrated strong performance in prior research [21, 22]. Our ablation experiments also demonstrated that setting the number of layers to 2 for both GraphSage and GCN can achieve good results. Please refer to Sect. "Ablation study" and Supplementary Figs. S1 and S2.

### Training and prediction

Denote the embedding of node *i* obtained from the graph neural network as ${H}_{i}$. To evaluate the strength of the association for a disease-gene pair (*d*_*i*_, *g*_*j*_), we employed cosine similarity (Eq. 9) as a measure:

9 $$\begin{array}{c}{score}_{ij}=\frac{{\widetilde{H}}_{i}{\cdot \widetilde{H}}_{j}}{\left|{\widetilde{H}}_{i}\right|\left|{\widetilde{H}}_{j}\right|}\end{array}$$

where ${\widetilde{H}}_{i}=[{H}_{i}||{H}_{i}^{node}]$, ${H}_{i}^{node}$ represents the node embedding obtained from node2vec and $|{\widetilde{H}}_{i}|$ is the norm of${\widetilde{H}}_{i}$.

During the training phase, negative samples were randomly selected from all unconnected pairs between diseases and genes. Due to the fact that the connected gene-disease pairs are significantly less than the unconnected gene-disease pairs, we set the number of negative samples to be *p* times the number of positive samples. To learn the parameters, the margin loss function was adopted, defined by Eq. 10:

10 $$\begin{array}{c}Loss\left({y}_{ij},{\widehat{y}}_{ij}\right)=Max\left(\text{0,1}-{\widehat{y}}_{ij}\cdot {y}_{ij}\right)\end{array}$$

where ${\widehat{y}}_{ij}={score}_{ij}$, and ${y}_{ij}$ represents the true relationship between gene node *i* and disease node *j*. Specially, ${y}_{ij}=1$ if there exists an association between *i* and *j*, otherwise ${y}_{ij}=0$.

During the prediction phase, scores were solely computed for the associations between each disease and its candidate genes. Afterwards, the candidate genes were ranked for each disease based on their respective scores.

## Results

### Datasets

The heterogeneous module network consists of two types of nodes that represent genes and disease, two types of links corresponding to disease-gene associations and protein–protein interactions, and one type of modules (protein complexes). The disease-gene associations and 213,888 protein–protein interactions were downloaded from the literature [23], which sourced the data from the DisGeNet [24] database. A total of 2822 protein complexes were collected from Human Protein Reference Database [25].

In accordance with the experimental methodology of the prior research [23], the disease-gene associations were classified into two distinct groups. The first group, denoted as the internal dataset, contained 130,820 disease-gene associations involving 13,074 diseases and 8947 genes, which was used for cross validation. The second group comprised 10,066 disease-gene associations involving 1186 diseases and 2552 genes. Termed as the external dataset, this group was collected from DisGeNet that integrated animal model data, which was used to assessment the capacity to discover new candidate associations.

### Experimental setting

We adopted the experimental settings proposed by Yang et al. [23]. To validate the effectiveness of our method, we conducted a tenfold cross validation on the 130,820 curated associations. Additionally, we used 10,066 associations from animal model as an external dataset for each fold. The parameter *l* in graph data augmentation is set to 10, resulting in a total of 243,379 newly added interactions. The hyperparameters were tuned with the help of cross validation. Specially, for the node2vec, we set the window size, the walk length, the number of walks, the in–out parameter, the embedding size and the iteration number to 5, 64, 10, 0.3, 128 and 10, respectively. For GAT, we set the size of hidden units for *GAT* to (256, 128), and the number of heads in multi-head attention to 2. The learning rate, epoch number and size of hidden units for *GCN* and *GraphSage* were set to 0.0009, 10 and (128, 64, 8), respectively. Moreover, the number of negative samples was set to be 50 times ($p=50$) greater than the number of positive samples.

In the experiments, Precision, Recall, F1-score (F1) and Association Precision (AP) were employed to evaluate the performance of gene prioritization. Denote the true pathogenic genes of the disease *d* in the test set as *T(d)*, and record the top *i* genes with the highest predicted probabilities for the disease d as ${P}_{i}(d)$. Precision, Recall, F1-score in Top@i can be defined as follows:

11 $$\begin{array}{c}Prec=\frac{1}{|D|}\sum_{d\in D}\frac{\left|T(d)\bigcap {P}_{i}(d)\right|}{\left|{P}_{i}(d)\right|} \end{array}$$

12 $$\begin{array}{c}Recall=\frac{1}{|D|}\sum_{d\in D}\frac{\left|T(d)\bigcap {P}_{i}(d)\right|}{\left|T(d)\right|} \end{array}$$

13 $$\begin{array}{c}F1=\frac{1}{|D|}\sum_{d\in D}\frac{2\left|T(d)\bigcap {P}_{i}(d)\right|}{\left|{P}_{i}(d)\right|+\left|T(d)\right|} \end{array}$$

To assess the overall performance, the association precision (AP) is defined as follows:

14 $$\begin{array}{c}AP=\frac{{\sum }_{d\in D}\left|T(d)\bigcap {P}_{k}(d)\right|}{\sum_{d\in D}\text{min}(\left|{P}_{k}\left(d\right)\right|,10)} \end{array}$$

Here, *D* is the disease set and *k* is set as the number of true pathogenic genes in the test for each disease. If the number of pathogenic genes for a certain disease is greater than 10, then set *k* as 10. The Eq. 14 imposes restrictions the list length of candidate genes, focusing solely on the top 10 candidate genes for each disease. This is because the exploration of gene-disease associations is essentially a ranking problem, and during cell experiments, animal model studies, and clinical trials, candidates are typically selected from the top-ranked genes. Additionally, AUC was utilized to evaluate the performance.

### Performance comparisons with state-of-the-art methods

**Fig. 2:**
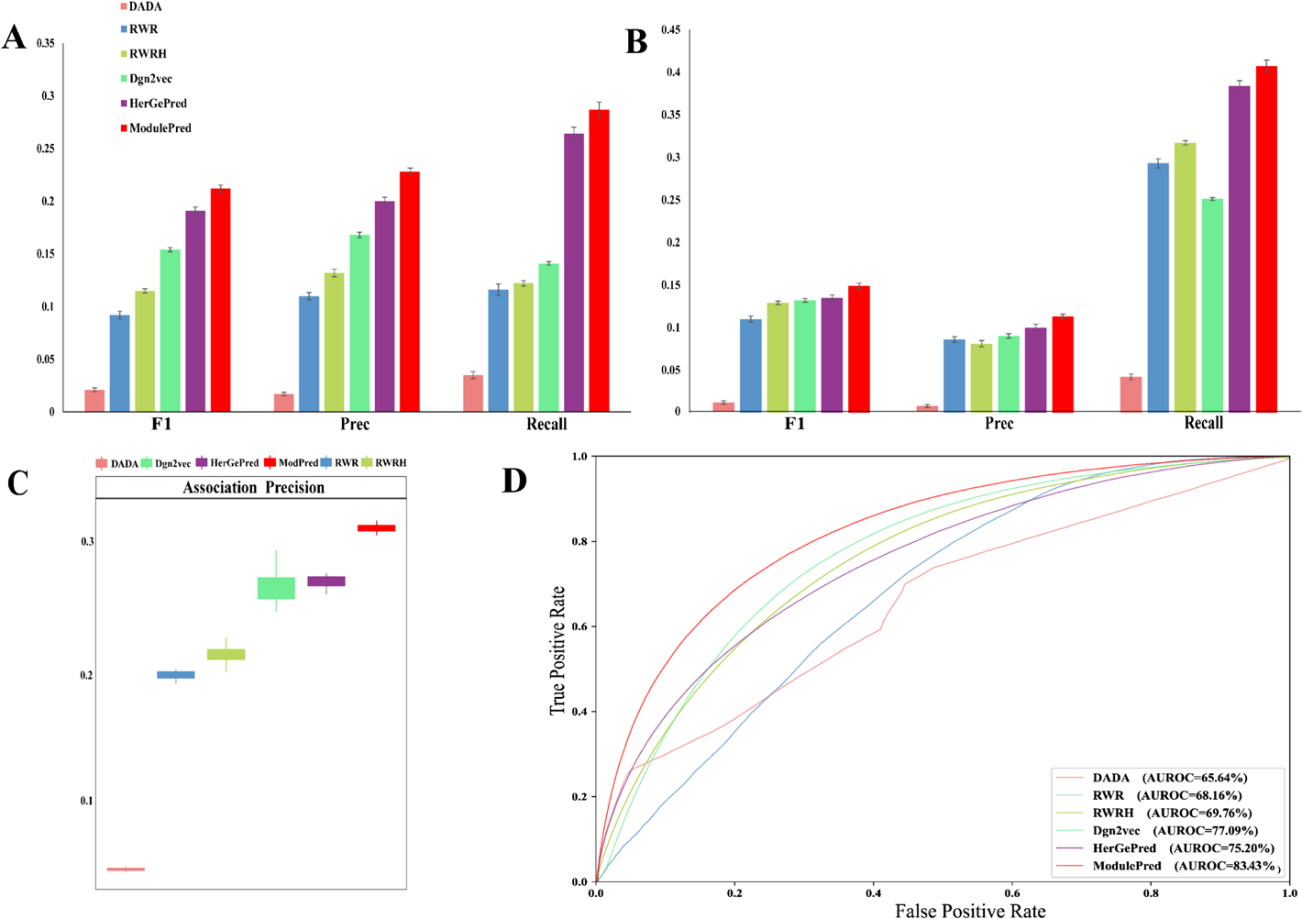
Cross validation performance comparison with state-of-the-art methods on the internal dataset. **A** The average F1, Precision and Recall of Top-3 predicted genes. **B** The average F1, Precision and Recall of Top-10 predicted genes. **C** AP performance. **D** ROC curves for disease gene prediction. Error bars represent the distribution of tenfold cross validations

To validate the superiority of our approach, we compared ModulePred with three state-of-the-art methods including DADA [26], RWR [27], RWRH [28], Dgn2vec [29] and HerGePred [23]. As depicted in Fig. 2, our approach demonstrated superior performance compared to these competitive methods. In terms of Top@3, ModulePred exhibited the highest Precision, Recall and F1 (Fig. 2A). Similarly, for Top@10, ModulePred significantly outperformed the other methods across the three metrics (Fig. 2B). When evaluating the overall performance using the Association Precision (AP), HerGePred outperformed the other baseline methods. However, our approach, ModulePred, showed remarkable improvement over HerGePred, with an increase of approximately 4 percentage points in AP (from 0.259 to 0.306; Fig. 2C) and 7 percentage points in AUC (from 0.752 to 0.834; Fig. 2D).

**Fig. 3:**
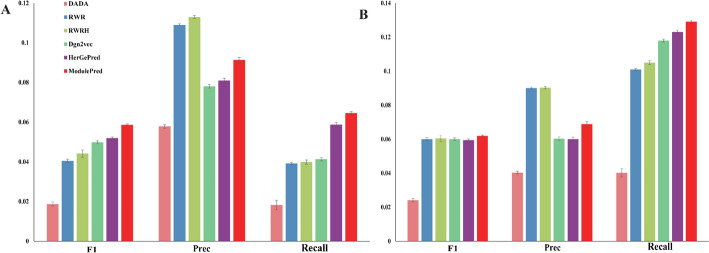
Performance comparison with state-of-the-art methods on the external dataset. **A** The average F1, Precision and Recall of Top-3 predicted genes. **B** The average F1, Precision and Recall of Top-10 predicted genes. Error bars represent the distribution of tenfold cross validations

To evaluate the capability for discovering new disease genes, we further assess the performance on the external dataset, as depicted in Fig. 3. In the Top@3 scenario, ModulePred outperformed other methods in terms of F1 and Recall, despite its Precision being lower than that of RWR and RWRH (Fig. 3A). Moreover, the performance of the methods in the Top@10 scenario was found to be similar to that in the Top@3 scenario (Fig. 3B). It is important to note that the performance on the external dataset in Fig. 3 was notably lower than that on the internal dataset in Fig. 2. This discrepancy arises from the fact that both the external and internal datasets were evaluated using the same prediction results. For example, assume that disease $d$ is associated with genes ${g}_{1}$, ${g}_{2}$, ${g}_{3}$, ${g}_{4}$ and ${g}_{5}$ in the internal dataset, and with genes ${g}_{6}$ and ${g}_{7}$ in the external dataset. In a fold of cross-validation, the training set includes two gene-disease associations ${(d,g}_{1})$ and ${(d,g}_{2})$, while the test set includes ${(d,g}_{3})$, ${(d,g}_{4})$ and ${(d,g}_{5})$. An algorithm predicts the top 3 candidate genes most likely associated with disease d as ${g}_{3}$, ${g}_{4}$ and ${g}_{5}$. In the Top@3 scenario, the algorithm achieves 100% precision, recall and F1 score in predicting disease $d$. Since the top 3 candidate genes have no intersection with the external dataset, the algorithm completely fails to discover new genes in the external dataset, leading to a bias in its performance on the external dataset.

### Ablation study

We compared the proposed ModulePred method with three ablations, namely GNN-M, GNN^*^ and GNN. Theses variants were compared as follows:

1. GNN^*^-M is the complete ModulePred method which uitlizes the augmented protein interaction network and applies graph representation with module information.
2. GNN-M is an ablation of ModulePred that applies graph embedding solely on the original protein interaction network.
3. GNN^*^ is an ablation of ModulePred that uses the augmented protein interaction network without modules and performs graph embedding using the traditional node2vec approach.
4. GNN is an ablation of GNN^*^ that uses the original protein interaction network without protein complexes.

**Fig. 4:**
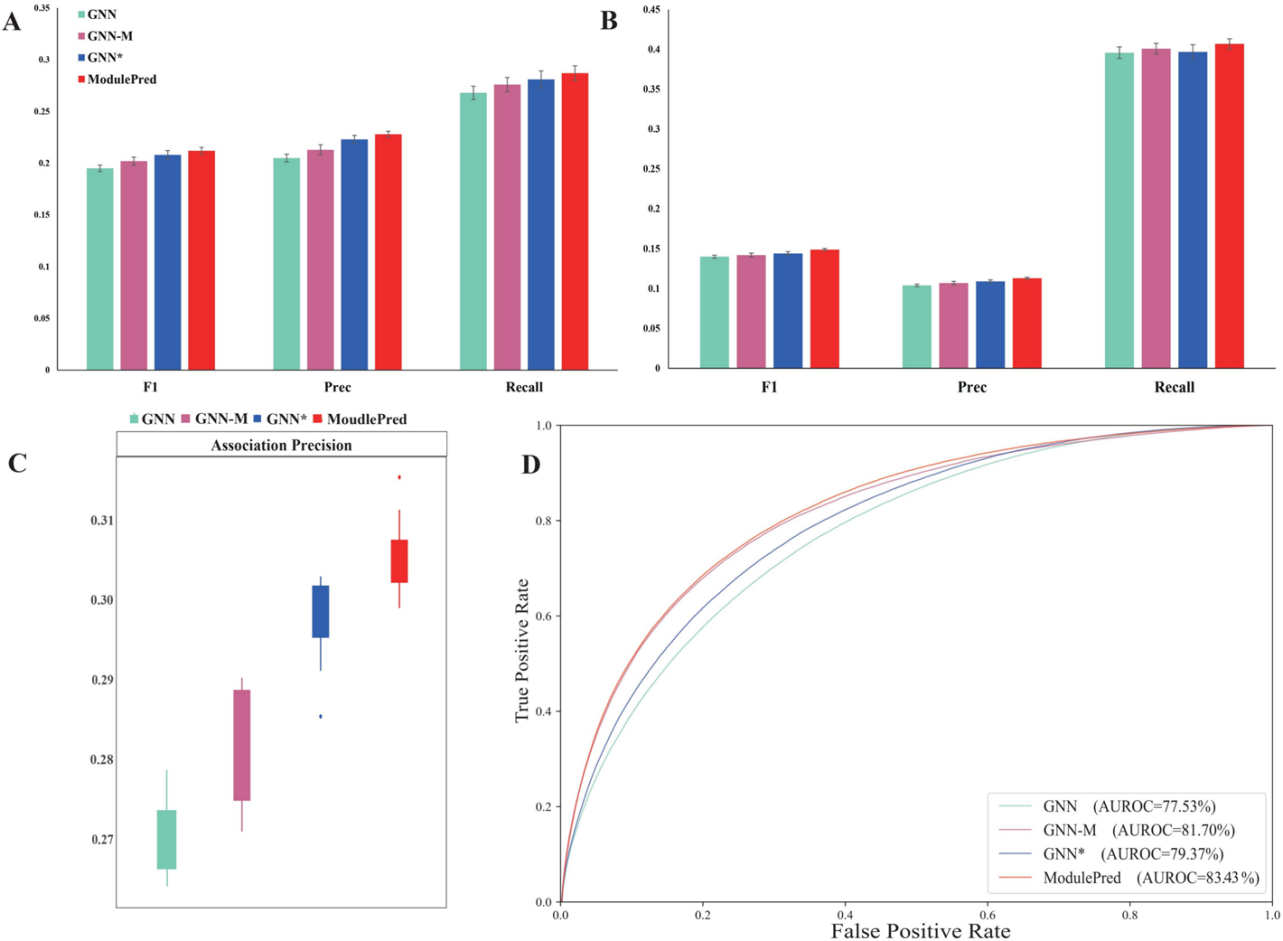
Cross validation performance comparison with three ablations on the internal dataset. **A** The average F1, Precision and Recall of Top-3 predicted genes. **B** The average F1, Precision and Recall of Top-10 predicted genes. **C** AP performance. **D** ROC curves for disease gene prediction. Error bars represent the distribution of tenfold cross validations

As depicted in Fig. 4, the incorporation of protein complexes allowed GNN-M to surpass GNN in all the evaluation metrics. Similarly, GNN^*^ utilzied the augmented protein–protein interaction network to investigate the connections between diseases and genes, resulting in significant notable enhancements across all evaluation metrics compared to GNN. Notably, the impact of data augmentation had a greater impact on the AP index compared to module information (Fig. 4C). ModulePred, which integrated both module information and augmented protein interactions, made substantial progress when compared to GNN^*^ and GNN-M (Fig. 4C).

**Fig. 5:**
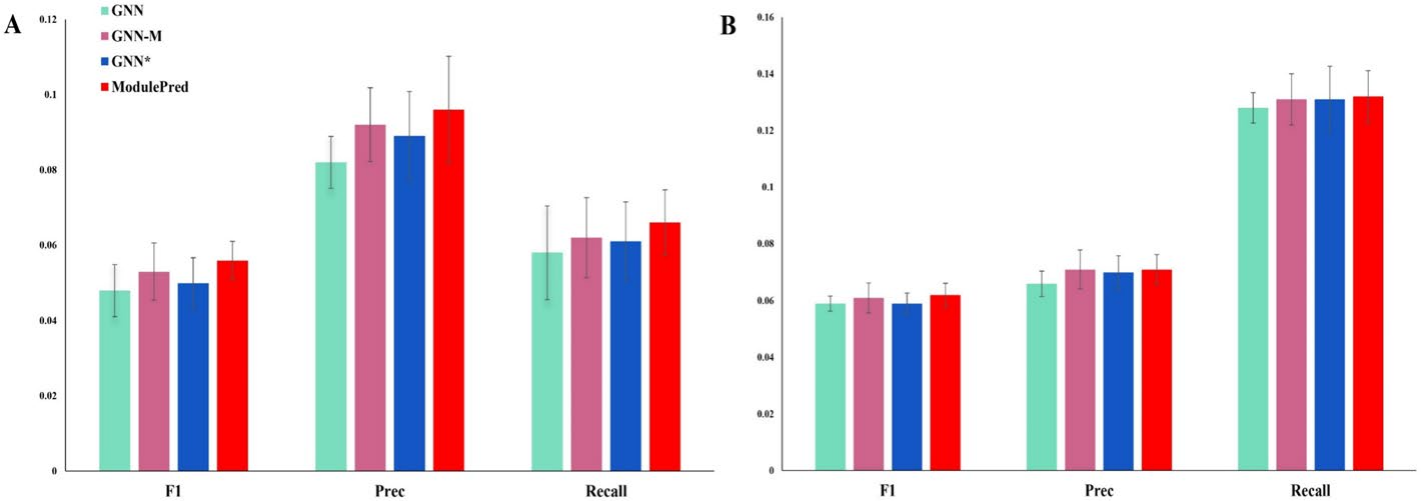
Performance comparison with three ablations on the external dataset. **A** The average F1, Precision and Recall of Top-3 predicted genes. **B** The average F1, Precision and Recall of Top-10 predicted genes. Error bars represent the distribution of tenfold cross validations

Furthermore, we performed an analysis on the external dataset (Fig. 5), once again confirming the superiority of ModulePred over three ablations. This reinforced the potential of our approach in uncovering novel disease-gene associations. Both GNN-M and GNN^*^ consistently exhibited better performance than GNN. However, GNN-M outperformed better than GNN^*^ on the external dataset, showcasing a deviation from their performance on the internal dataset.

We further conducted three additional ablation experiments. Figure S1 indicates that the network structure of ModulePred (utilizing GAT for processing heterogeneous networks, GraphSage for processing gene-disease associations, and GCN for processing protein–protein interactions) can achieve good performance. Figure S2, suggests that setting the number of GAT layers to 1, GraphSage to 2 and GCN to 2 in ModulePred is an optimal parameter configuration. Moreover, Figure S3 demonstrates that setting the number of *l* in graph data augmentation to 10 can achieve optimal performance.

### Case study

To further elucidate the biological insights of our approach, we conducted two case studies in order to identify disease genes related to hypothyroidism and Idiopathic Pulmonary Arterial Hypertension (IPAH). The predicted genes were ranked based on their scores (refer to Eq. 9 for details). Furthermore, we manually searched published biomedical literature to obtain final confirmations.

IPAH is a progressive and potentially life-threatening condition characterized by elevated blood pressure in the pulmonary arteries without any discernible underlying cause, requiring thorough investigation and management from a medical perspective [17]. Among the top 10 genes predicted by ModulePred (Table 1), an impressive 6 associations were substantiated by previous publications, supported by their corresponding PubMed Unique Identifier (PMID). For instance, the top-ranked gene MIR204 has been reported to exhibit abnormal expression in relation to the onset and progression of IPAH [30].

**Table 1:** Top 10 predicted genes for IPAH

Rank	Gene	Reference
1	MIR204	PMID: 30,854,934
2	CBLN2	PMID: 27,770,446
3	OTSC1	NA
4	EIF2AK4	PMID: 31,711,431
5	ENG	PMID: 30,312,106
6	PYCR1	NA
7	RTEL1	PMID: 30,523,160
8	LBR	NA
9	B3GAT3	NA
10	TGFBR3	PMID: 11,282,888

Hypothyroidism is a multifaceted endocrine disorder characterized by diminished production or action of thyroid hormones, resulting in a variety of physiological disruptions that necessitate investigation and management from an endocrinological perspective. Recent studies have identified several genes associated with hypothyroidism [31–33]. As presented in Table 2, our ModulePred achieved high prediction accuracy rates of 100%, 80%, 86% for the top 2, top 5 and top 7 genes, respectively. For instance, OTX2 Mutations have been linked to developmental abnormalities in both the central nervous system and the thyroid, resulting in hypothyroidism [34]. Similarly, defects in GLI2 can disrupt normal thyroid development and function, potentially leading to reduce thyroid hormone levels [35].

**Table 2:** Top 10 predicted genes for Hypothyroidism

Rank	Gene	Reference
1	LHX3	PMID: 12,244,277
2	OTX2	PMID: 26,416,826
3	GALE	NA
4	MAGEL2	PMID: 33,570,896
5	BRAF	PMID: 21,512,141
6	GLI2	PMID: 25,484,916
7	FANCB	PMID: 28,588,452
8	CDKN1C	NA
9	NDST1	NA
10	PAH	NA

## Conclusion

In this article, a deep learning framework called ModulePred is presented for predicting disease-gene associations. ModulePred achieves competitive predictive performance by employing graph augmentation on the protein interaction network and graph embedding for the heterogeneous module network. Experimental results on the DisGeNet dataset substantiate the efficacy of ModulePred in discovering disease-gene associations. Furthermore, the ablation study highlights the greater impact of graph augmentation on the performance of ModulePred compared to the graph embedding for the module network.

## Supplementary Information

Supplementary Material 1.

## Data Availability

All datasets and code in this work are available at https://github.com/qdu-bioinfo/ModulePred. All other relevant data is available upon request.

## Abbreviations

SNPs: Single nucleotide polymorphisms
GCN: Graph convolutional network
PPI: Protein–Protein interaction
GAT: Graph attention network
F1: F1-score
AP: Association precision
IPAH: Idiopathic pulmonary arterial hypertension
PMID: PubMed unique identifier
