# Supplementary material for: A deep learning framework for predicting disease-gene associations with functional modules and graph augmentation
Source: BMC Bioinformatics. 2024 Jun 14;25:214. doi: 10.1186/s12859-024-05841-3 (PMC11549817; doi:10.1186/s12859-024-05841-3)
Supplement: Supplementary file 1 — Supplementary Material 1. [file 12859_2024_5841_MOESM1_ESM.docx]

**Supplementary materials**


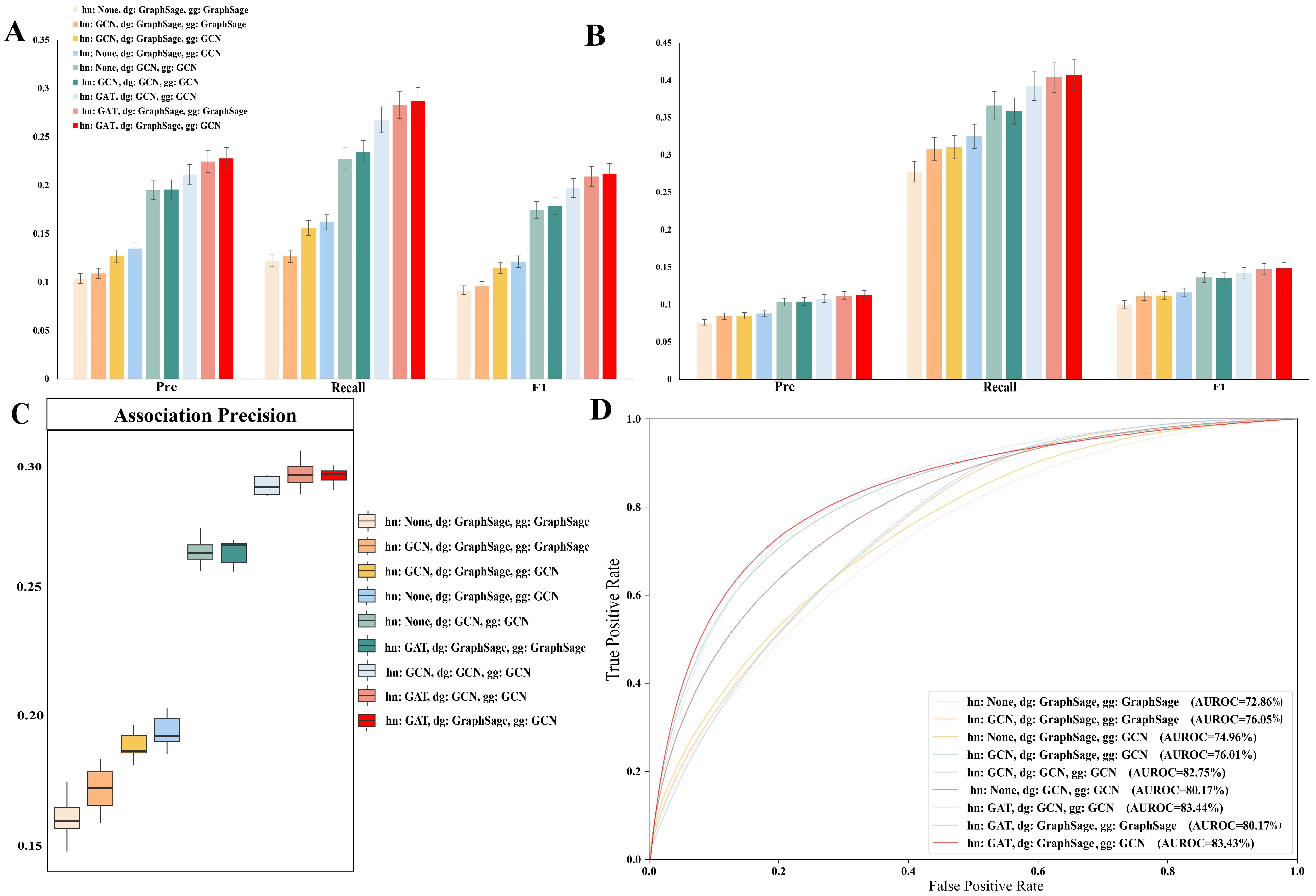


**Figure S1: Performance comparison with 8 ablations on the internal dataset. (A)** Top-3 predicts the average F1, Precision, and Recall of genes. **(B)** The average F1, Precision, and Recall of the top 10 predicted genes. **(C)** AP performance. **(D)** ROC curve for disease gene prediction. The error bar represents the distribution of 10 times cross validation.

"hn: *, dg: &, gg: ^" represents the utilization of * for processing heterogeneous networks, & for processing gene-disease associations, and ^ for processing protein-protein interactions. In the case of ModulePred, hn: GAT, dg: GraphSage, gg: GCN. As shown in **Figure S1**, ModulePred performs the best overall, indicating that utilizing GAT for processing heterogeneous networks, GraphSage for processing gene-disease associations, and GCN for processing protein-protein interactions can effectively explore gene-disease associations.

**
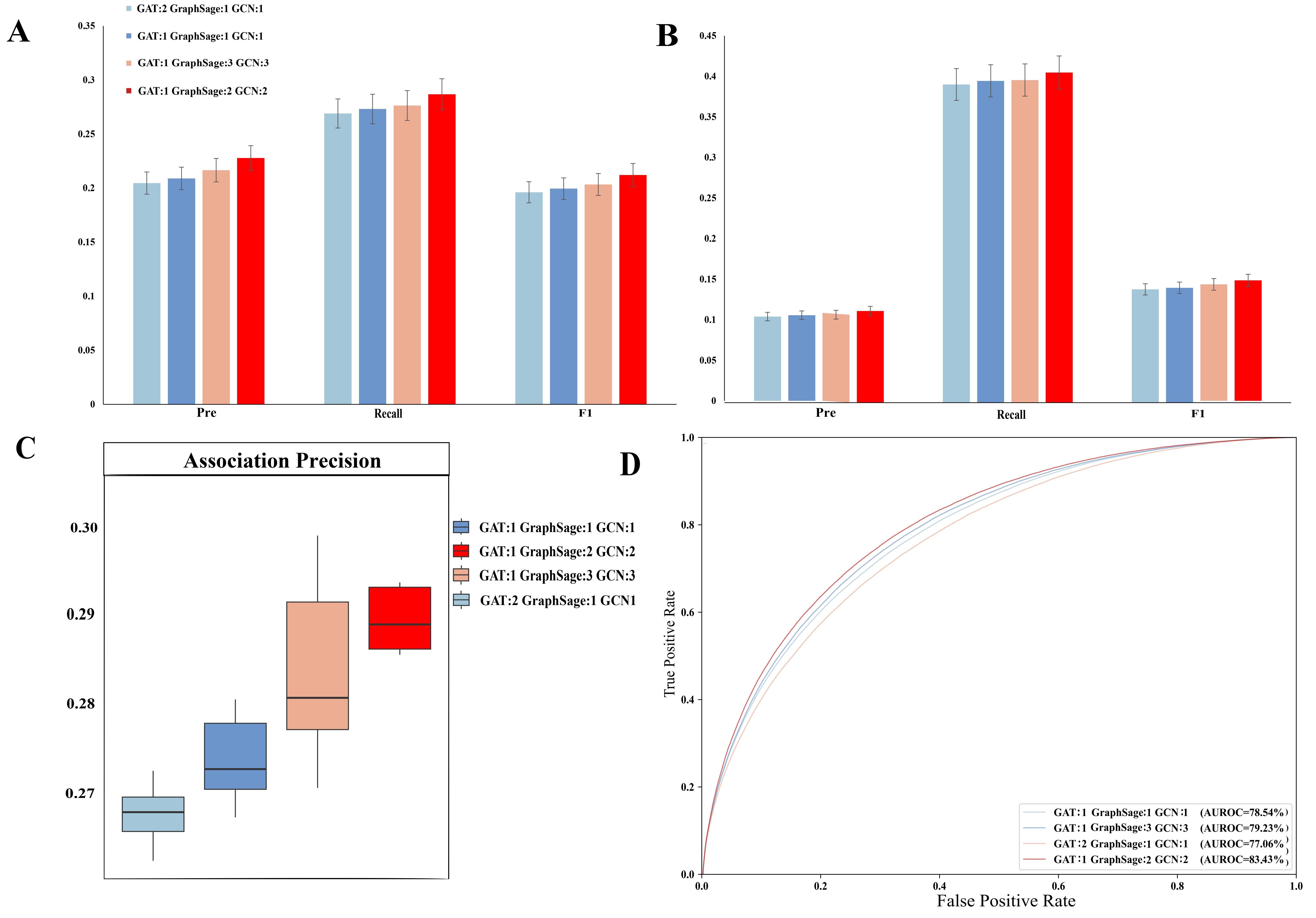
**

**Figure S2: Performance comparison with 3 ablations on the internal dataset. (A)** Top-3 predicts the average F1, Precision, and Recall of genes. **(B)** The average F1, Precision, and Recall of the top 10 predicted genes. **(C)** AP performance. **(D)** ROC curve for disease gene prediction. The error bar represents the distribution of 10 cross validations.

"GAT: x, GraphSage: y, GCN: z" signifies setting the number of GAT layers in ModulePred to x, GraphSage layers to y, and GCN layers to z. As illustrated in **Figure S2**, setting the number of GAT layers to 1, GraphSage layers to 2, and GCN layers to 2 yields optimal performance.





**Figure S3: Performance comparison with the increase in the number of newly added interactions in graph data augmentation. (A)** Top-3 predicts the average F1, Precision, and Recall of genes. **(B)** The average F1, Precision, and Recall of the top 10 predicted genes. **(C)** AP performance. **(D)** ROC curve for disease gene prediction. The error bar represents the distribution of 10 times cross validation.

"*l*=*x*" represents ModulePred assigns the parameter *l* in graph data augmentation to *x*, indicating *x* new edges are constructed for each protein. As illustrated in **Figure S3**, setting *l* to 10 achieved good prediction performance.
